# Effect of the cleaning process on physical properties for different malting barley seed varieties

**DOI:** 10.1002/fsn3.1609

**Published:** 2020-05-01

**Authors:** Veronika Hetclova, Lucie Jezerska, Kristina Strbova, Rostislav Prokes, Jiri Zegzulka

**Affiliations:** ^1^ Department of Mining Engineering and Safety Faculty of Mining and Geology VSB – Technical University of Ostrava Ostrava Czech Republic; ^2^ ENET Centre VSB – Technical University of Ostrava Ostrava Czech Republic

**Keywords:** ANOVA, barley seeds, cleaning process, correlation analysis, physical properties

## Abstract

The aim of the study was to investigate the influence of the barley cleaning process in relation to physical properties. The knowledge of the range of changes in the physical parameters of processed material and their mutual relationships is required for the design and implementation of various technological processes. In this study were compared the input and output commodities in the primary postharvest cleaning process of undesirable components—occurring as admixtures of fine and coarse barley impurities as well as the barley component itself. An efficient cleaning process ensuring barley grain quality is a basic step in beer production. Therefore, seven bred varieties of brewing barley (Malz, Sebastian, Francin, KWS Irina1, KWS Irina2, Bojos, and Laudis) were tested for the qualitative assessment of the cleaning process. Physical parameters such as granulometry, bulk and tapped density, angle of repose, internal and wall friction angle, and flow functions were determined for all samples. In order to identify whether the barley variety or the sample cleaning significantly influences the determined physical properties, two‐way ANOVA was applied. The results imply that barley cleaning had the main influence on wall friction angle, while the barley variety had a significant effect on effective internal friction. Moreover, the mechanical postharvest cleaning process reduces the overall wall friction.

## Graphical abstract

An efficient cleaning process ensuring barley grain quality in beer production was studied. Seven varieties of barley were characterized before and after the process. Two‐way ANOVA results showed that the separation process and barley variety had the main influence on wall friction angle values.

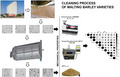

## 1 INTRODUCTION

Barley is one of the oldest and most widespread crops grown worldwide (Albini, Freire, & Freire, 2018; Idehen, Tang, & Sang, 2017). In the Czech Republic (the temperate zone), it is the second most important cereal crop. Barley is widely used for feeding purposes (approximately 65%), specially bred malting barleys are used in the brewing industry (33%) and only a small proportion is used for human consumption (2%) (Sullivan, Arendt, & Gallagher, 2013). According to the Central Institute for Supervising and Testing in Agriculture, seventy‐two barley varieties were registered in 2018 (Central Institute for Supervising & Testing in Agriculture, 2020). Due to the different harvesting periods, barley has varying chemical compositions, which is due to environmental conditions, especially soil, climate, weather conditions, nutrition, and variety, but also the method of cultivation, cleaning, and harvest time (Striegel & Zidkova, 1993).

The pursuit of an objective and comprehensive opinion on the malting quality of barley varieties has led to the development of different evaluation systems and procedures. At present, the malting quality of barley varieties is evaluated according to the “malting quality indicator,” which was created based on the requirements of the processing industry (Rhodes, 2008). The key indicators of malting barley quality are mainly germination and germination energy (Daneri‐Castro, Svensson, & Roberts, 2016). Germination is given as the percentage of germinated seeds over a specified period (6 days) under laboratory conditions. It is the quantity expressed as a percentage of all live cereals in the sample. The germination energy is expressed as the percentage of caryopses in the sample that germinate under natural conditions for 3 days at the time of determination. Causes of reduced germination may be due to disturbances in seed development, defects in fertilization, uneven maturation, weather conditions, harvest damage, poor postharvest treatment, and inappropriate storage (Rhodes, 2008). A very important feature is the content of nitrogenous substances—the protein and starch content (Balet, Gous, Fox, Lloyd, & Manley, 2020). Other important quality criteria are the physical characteristics, such as the proportion of grain above the 2.5 mm sieve, at a basic value of 80%, and the basic value of 20% at a size of 2.8 mm. Malting barley should not contain any waste—desiccated and undeveloped grains that fall through a sieve of 2.2 mm (Fastnaught, Berglund, Holm, & Fox, 1996; Izydorczyk & Dexter, 2005; Kosar & Prochazka, 2000).

In the cleaning process, dust, light, and metal particles are removed. The removed particles could be subject to further research (i.e., recycling them by torrefaction and ecological disposal). The sorting of barley grains by size is of technological importance for achieving uniform steeping and germination and obtaining a perfectly homogeneous malt (Chladek, 2007). It is evident that its detailed analysis will help us understand the basic principles and to find critical areas with the possibility of technical innovation. After the cleaning process, the barley seeds are stored in silos.

Therefore, the aim of the study was to determine the physical properties of various barley varieties in their raw state and in the postcleaning state (uncleaned and cleaned samples, respectively). This was mainly the bulk angle, bulk density, tapped density, internal and wall friction angle, and the resulting flow properties and granulometry, as well as to compare the possible effect of the cleaning process on these barley grains and their interconnection with physical factors (influences).

Information on the flow characteristics of barley grains has to be verified from the perspective of determining the capacity of the storage space, transport, emptying or filling silos and for the correct design of the cleaning facility (Aghajani, Ansaripour, & Kashaninejad, 2012). The magnitude of internal and wall friction angles is the basis for designing the correct construction of containers and other storage equipment (Vilche, Gely, & Santalla, 2003). Knowledge of the particle size distribution of the input barley is one of the primary properties for designing machinery for cleaning, sorting, separation, and storage (Kachru, Gupta, & Alam, 1994). Another important and interesting feature, often neglected, is the morphology of barley grains, which allows the particle surface to be calculated, which is an important aspect, for example, in the design of drying technology (Sologubik, Campañone, Pagano, & Gely, 2013). In addition, the friction parameters of barley seeds are important in order to design the storage structures and the selection of equipment used in the whole technological line (Hamdani et al., 2014; Tavakoli, Tavakoli, Rajabipour, Ahmadi, & Gharib‐Zahedi, 2009).

However, the information on the systematic determination of the basic physical properties of barley varieties currently available in literature has not been comprehensively processed. Therefore, the systematic characterization and comparison of individual varieties is a necessity, not only to achieve an optimum design of technological line parts but also to ensure sufficient product quality. The aim of the study was to investigate the possible influence of barley cleaning processes in relation to physical properties, including statistical data analysis, which proves the interconnection of the tested parameters.

## 2 MATERIALS AND METHODS

### 2.1 Test materials

**Figure 1:**
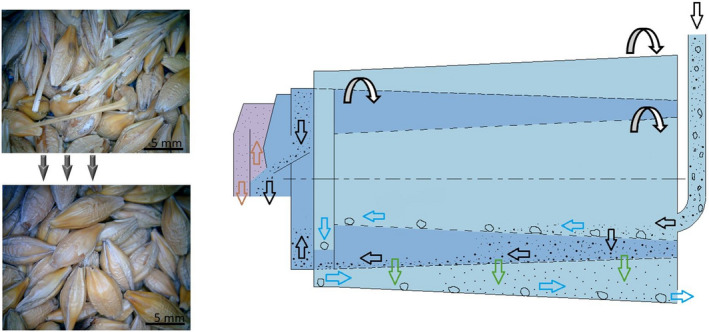
Barley seeds’ images and scheme of their cleaning. Left—sample of the Laudis variety—from uncleaned to cleaned. Right—diagram of the cleaning principle

The test material was malting barley (Figure 1). Seven varieties of specially bred brewing barley grown in the Czech Republic —Moravia—were selected. These barley samples were taken from hoppers–silos. The weight of uncleaned barley into the silo was 30 t for each variety. Then, the uncleaned barley was cleaned in a combi‐cleaner (Figure 1 right). The weight of one sample of both the uncleaned and the cleaned samples was 5 kg. These are the Malz, Sebastian, Francin, KWS Irina1, KWS Irina2, Bojos, and Laudis species. Initial inspection during the storage was performed in order to determine the basic parameters of the quality of the samples; following parameters were determined: humidity in the range of 13%–13.2%, overflow of the oversize fractions of 2.5 mm in the range of 91.6–95.5 wt. %, fall through of below‐sieve fractions of 2.2 mm from 4.5 to 7.3 wt. % and a germination total of 99%. The determined values were in agreement with the previously reported values (Fastnaught et al., 1996; Izydorczyk & Dexter, 2005).

Samples of seven barley varieties were collected at the time of silo delivery, in both their raw, uncleaned state containing impurities of clay, stones, grass blades, damaged grains, metal particles, dust, and barley material samples after the cleaning process.

For the cleaning process, the KDC 4000 combi‐cleaner (Kongskilde, Denmark) was used (Figure 1 right). It is a combined screen and aspiration cleaner with capacities of up to 40 t/h, commonly used for the cleaning of malting barley and seed. Motor outputs are 1.5 kW for the screen drum, 4 kW for the fan, and 0.75 kW for the auger. The material enters the cleaning device in the upper part of the machine (black arrow). In the first cylinder, the material mixtures are separated from coarse/large impurities. In order to obtain optimal efficiency, a cylinder screen with the correct slot/hole dimensions has to be used properly.

The inner cylinder has in its wider end round meshes with an 11 mm diameter—and the same mesh diameter is up to 2/3 of the cylinder length; in the last 1/3 of the cylinder length, the round mesh diameter is 9 mm. This is according to a standard recommendation, when at the beginning, the process capacity is increased, and afterward, optimal separation of coarser impurities is achieved, and simultaneously, the mesh capacity is decreased (finer meshes in the last 1/3).

In the outer cylinder, the remaining material particles fall through, with barley being separated from fine impurities. Thus, it is the slot/hole dimensions of the outer screen that determine the size of the impurities arrested. The size of oblong holes is 2.5 × 16.5 mm. This size is appropriate for the very few good grains removed. Subsequently, the fine impurities fall through (green arrow) and the finest particles are removed (brown arrow).

### 2.2 Methods

#### 2.2.1 Particle size distribution and particle shape

For measuring the size and shape of grains of free‐flowing bulk materials ranging from 30 µm to 30 mm, a CAMSIZER optoelectronic device was used, consisting of a planar light source, feeder for the measured material, two CCD cameras for generating images of the loose material, a cleaning unit, and a PC with an evaluation program. Particles of bulk material fall over the end edge of the vibrating trough, with rotation occurring during their fall. The rotating particles pass through the instrument through the measuring space—images are then generated using one or two CCD cameras, which are then analyzed. Granulometric analysis of the test material is generated automatically from the acquired images (Schwedes, 2003). After completion of the measurement, the results can be displayed in many formats, which are directly available for use. The measurements were made in a single cycle. The following values were measured: d_10_, d_50_, and d_90_, and further, SPHT_3_ (sphericity, Equation 1) and b/l_3_ (ratio of width to length, Equation 2):

(1) $${\text{SPHT}}_{3}=\frac{4\pi A}{U^{2}}$$

(2) $$\frac{b}{l_{3}}=\frac{\min(x_{c})}{\max(x_{\text{Fe}})}$$

#### 2.2.2 Bulk and tapped densities

A sample of barley of the determined weight was poured into the measuring cylinder, and the value of the apparent volume was subtracted. The bulk density was established as the share of weight and apparent volume (Mellmann, Hoffmann, & Fürll, 2014). The result is the average value of ten measurements. Subsequently, the sample was subjected to tapping vibrations (amplitude 2 mm, 250 strokes per minute) at rates of 10x, 500x, and 750x. The apparent volumes were subtracted for each given tapping number and settling of the sample. The tapped density was determined as the ratio of the sample weight to the sample volume after the last tapping at 750x if no change above 2 ml occurred. Otherwise, 1,250 taps were performed. The result is an average of 10 measurements.

#### 2.2.3 Angle of repose

The barley sample of the specified weight was poured into a hollow cylinder, from where it was evenly poured out onto a horizontal surface into a cone shape (ASTM International, 2000). Upon completion of the pouring, the cone was divided into 8 parts, of which the angle was subtracted from each location using an enclosed calibrated spirit level to one decimal place. The bulk angle was determined as the average of the 8 parts. The measurement was repeated 5 times.

#### 2.2.4 Angle of internal friction and wall friction

The principle of measuring the shear properties of bulk materials consists in weighting the bulk material with a normal load of a preset size and then shifting layers of the bulk material in a direction perpendicular to the normal load. A shear stress is generated between the particles due to friction, which is detected. Specifically, the force needed to overcome the shear forces between the particles is measured by the action of the normal force (Zegzulka, 2013). These forces are further converted to stress (Scieszka & Adamecki, 2013). The measurement of the internal and wall friction angles was performed on a Schulze (Schulze Ring Shear Tester RST‐01, Wolfenbuttel, Germany) rotary shear machine.

**Figure 2:**
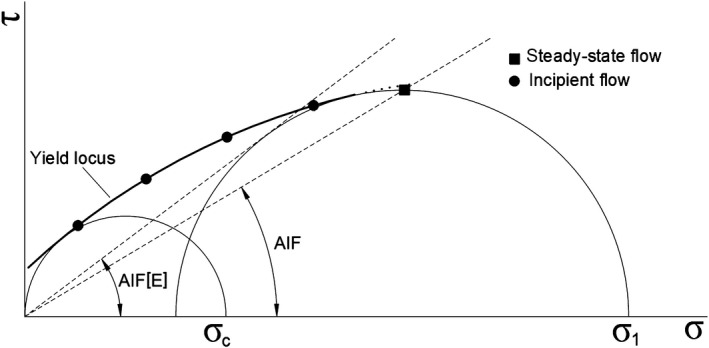
Schematic output of yield locus obtained from the Schulze Ring Shear Tester RST‐01

Figure 2 shows the schematic output with a basic description of the parameters related to the angle of internal friction. There are marked the effective angle of internal friction AIF[E], angle of internal friction in steady‐state flow AIF, major principal stress σ_1_, and unconfined yield strength σ_c_. Measurements made to determine how the cleaning process affects the frictional parameters of barley are focused primarily on the effective internal friction angle. The major part of the Schulze output data is just in connection with just this value.

The wall friction angle is a measure of the sliding friction at the grains/wall interface. Stainless steel was used as a contact material. This material represents the wall surface of the hopper, silos, conveyor, etc. It is determined by shear cell testing where the grains under the load slide across a pad representing the equipment's wall surface, and it is expressed in degrees.

The measurement was performed in three cycles. The result is an average value.

#### 2.2.5 Flow properties of barley grains (flowability)

The Hausner ratio (HR) and Carr's Index (CI) were used to define the flow properties of barley grains from tapped density (ρ_tp_, Equation 4) and bulk density (ρ_b_, Equation 5) (ASTM International, 2000). The Hausner ratio (HR) uses Equation 3:

(3) $$\text{HR}=\frac{\rho_{\text{tp}}}{\rho_{b}}$$

(4) $$\rho_{\text{tp}}=\frac{m}{V750}$$

(5) $$\rho_{b}=\frac{m}{\text{Vzd}}$$

Carr's Index (CI) can be obtained using the Equation (6).

(6) $$\text{CI}=\frac{\rho_{\text{tp}}-\rho_{b}}{\rho_{\text{tp}}}$$

The smaller the value of HR and CI, the better the flow properties of the material. Inclusion of the tested material in the flow modes according to individual evaluation criteria is shown in Table 1.

**Table 1:** Flow character of material according to individual evaluation criteria

Flow characteristics	Angle of repose (º)	Hausner ratio (–)	Carr's index (%)
Excellent	<25–30	1.00–1.11	1–10
Good	31–35	1.12–1.18	10–15
Adequate	36–40	1.19–1.25	16–20
Average	41–45	1.26–1.34	21–25
Poor	46–55	1.35–1.45	26–31
Very poor	56–65	1.46–1.59	32–37
Extremely poor	˃66	˃1.6	˃38

### 2.3 Statistical analysis

A two‐way analysis of variance (ANOVA) was used to identify whether the barley variety or the sample cleaning significantly influences both the angle of internal friction (AIF [E]) and the angle of wall friction (WFA), as well as whether the effect of the variety on the AIF [E] or WFA depends on the cleaning and vice versa. The relationships between all the measured physical characteristics were assessed by correlation analysis, where the Pearson correlation coefficient was determined. For significantly correlated pairs, a linear regression analysis was performed. All the statistical analyses were performed in statistical software (Harrell, 2015). The significance level was *p* = .05 for all the analyses.

## 3 RESULTS AND DISCUSSIONS

### 3.1 Particle characterization

**Figure 3:**
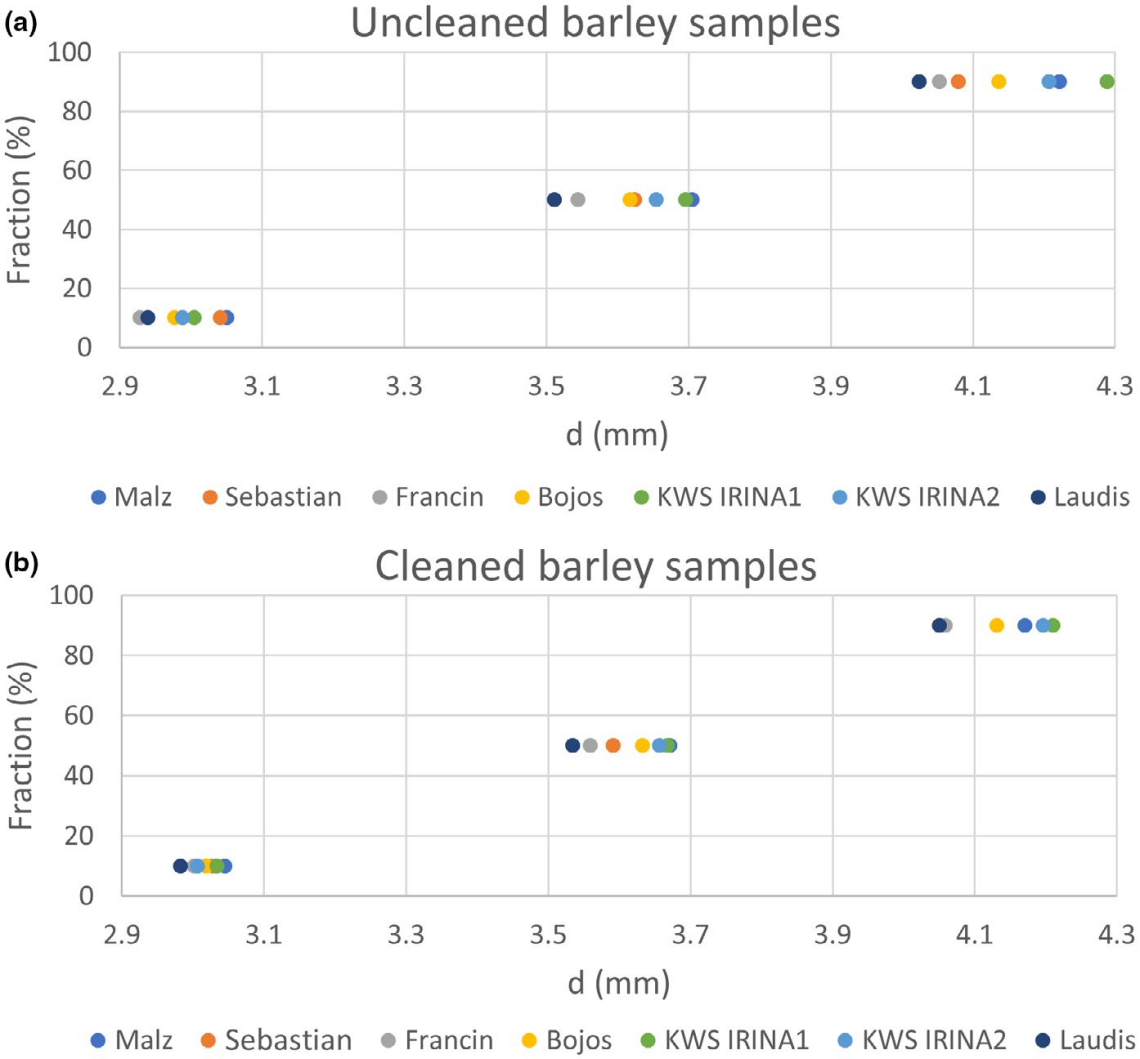
Particle size distribution. Upper—uncleaned barley samples. Lower—cleaned barley samples

The particle size distribution values for the measured barley samples in the raw state and after cleaning are summarized in Figure 3. This distribution was made with the aim of data orientation.

And greater dispersion of the measured parameters (fraction—d_10_, d_50,_ and d_90_) is evident from the graph for uncleaned samples. The most significant reduction is logical for the larger grains. When determining the distribution of the sizes of the grains of the uncleaned sample, the impurities that were removed by the cleaning process were also determined. A relatively larger shift to lower values after the cleaning process occurred for the KWS Irina1 sample, which would correspond to the more significant amount of impurities in relation to the other samples. The Bojos and KWS Irina2 samples appear to be identical before and after cleaning from the perspective of the size of the grains.

SPHT₃ symmetry (sphericity) values for uncleaned samples fluctuate in a greater range, by about 2.6%, compared with the samples after cleaning (Table 2). The greater dispersion in the case of uncleaned material is likely to be due to the variety of impurities mixed in. Increased value after cleaning suggests approaching a symmetrical grain shape. The edges of the barley grains are gently abraded by cleaning. Thus, the value of sphericity increases. The width‐to‐length ratios of b/l_3_ are found to be between 0.492 and 0.528 for uncleaned specimens and between 0.509 and 0.533 for specimens after cleaning, which is 6.9% greater than after the specimen cleaning.

**Table 2:** SPHT_3_ symmetry (sphericity) and b/l_3_ (width/length ratio) values for barley samples

Barley	SPHT_3_	b/l_3_
	Uncleaned/cleaned	Uncleaned/cleaned
Malz	0.685/0.706	0.492/0.509
Sebastian	0.714/0.729	0.517/0.521
Francin	0.725/0.739	0.525/0.533
KWS Irina1	0.705/0.731	0.507/0.531
KWS Irina2	0.712/0.713	0.509/0.511
Bojos	0.730/0.733	0.528/0.530
Laudis	0.722/0.724	0.524/0.527

It is evident that the values characterizing the grain shape, in particular SPHT_3_ (sphericity rate) and the width‐to‐length ratio b/l₃ for the samples after cleaning, are greater than those measured before cleaning. This may be due to the cleaning of a greater number of nonhomogeneous or otherwise inadequate grains (fragments, fractions) and impurities and the remaining quality grains of approximately the same width‐to‐length ratio. In this respect, the cleaning process improved homogeneity.

### 3.2 Effect of the cleaning process on seed flowability

**Figure 4:**
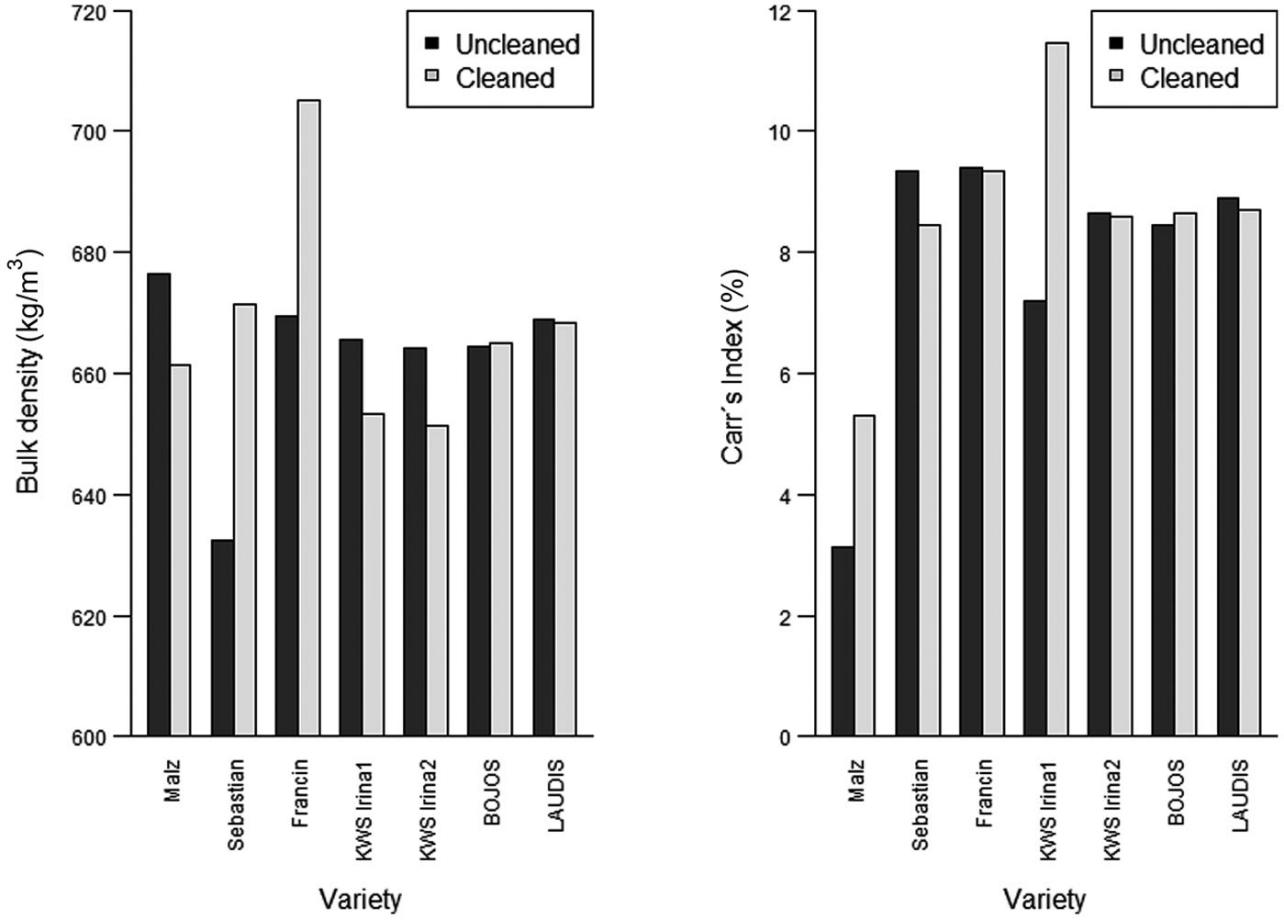
Effect of the cleaning process on the flowability characteristics. Left—bulk density dependence on the cleaning process in different barley varieties. Right—Carr's Index (CI) dependence on the cleaning process in different barley varieties

**Figure 5:**
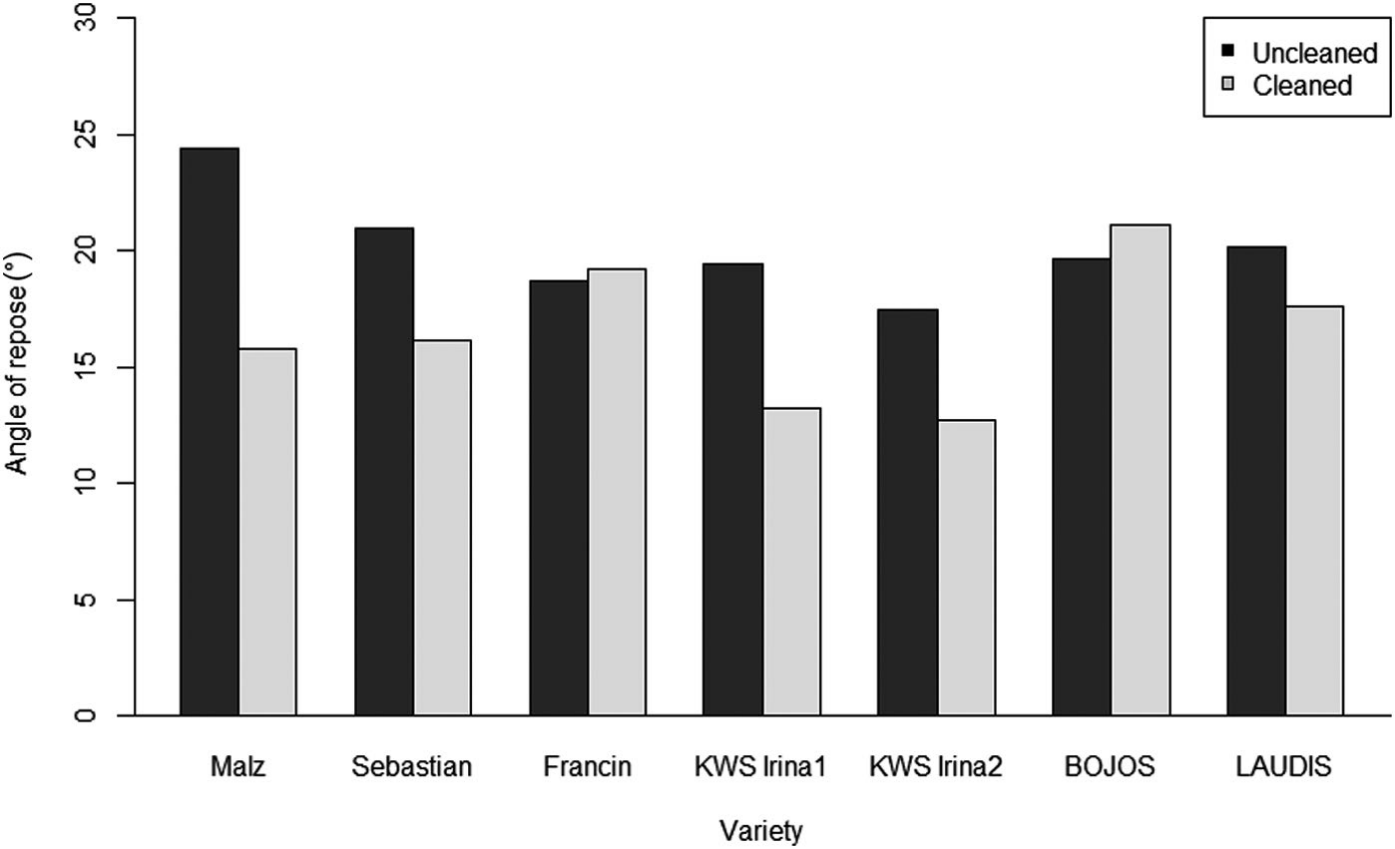
Angle of repose (AOR) depending on the cleaning process in different barley varieties

The dependence of the bulk density, Carr's index (CI), and the angle of repose (AOR) on the cleaning process in different barley varieties is shown in Figures 4 and 5, respectively. The classification in the individual flow modes according to values HR, AOR, and CI is shown in Table 3.

**Table 3:** Characteristic values for flowability parameters and flowability character evaluation

Barley	ρ_TP_ (kg/m^3^)	HR (–)	Flowability according HR	Flowability according AOR	Flowability according CI
	Uncleaned/cleaned	Uncleaned/cleaned	If change»	Same character for uncleaned and cleaned ↑ Increase or ↓ decrease of AOR	If change»
Malz	698.3/698.2	1.03/1.06	Excellent	Excellent/cleaned ↓35%	Excellent
Sebastian	697.7/733.2	1.11/1.10	Excellent	Excellent/cleaned ↓23%	Excellent
Francin	738.9/778.0	1.10/1.11	Excellent	Excellent/cleaned ↑ 3%	Excellent
Bojos	725.5/727.9	1.10/1.10	Excellent	Excellent/cleaned ↑ 7%	Excellent
KWS Irina1	717.3/738.1	1.10/1.23	Excellent» Adequate	Excellent/cleaned ↓ 32%	Excellent» Good
KWS Irina2	727.1/713.1	1.10/1.10	Excellent	Excellent/cleaned ↓ 27%	Excellent
Laudis	734.3/731.6	1.10/1.10	Excellent	Excellent/cleaned ↓ 13%	Excellent

A considerable difference in bulk density values between uncleaned and cleaned samples is evident in the case of the Sebastian and Francin varieties, while for the rest of the varieties, the difference was not significant or negligible. The bulk densities of barley varieties were close to values reported for hulled barley cultivars from high altitude Himalayan regions (Hamdani et al., 2014).

It can be said that the cleaning of the tested materials does not in any significant way influence their flow properties (Figure 4, Table 1). In all cases except KWS Irina1, the flow character was defined as excellent according to their CI (Table 3).

The angle of repose (AOR) values show a change in the flow after cleaning for all the samples and in the framework of an excellent character (Figure 5, Table 3). Despite the fact that the changes of the angle of repose values for cleaned and uncleaned samples achieved up to 35%, according to the flowability AOR assessment (Table 1, Table 3), all samples belong to the group “Excellent.”

The values of the Hausner ratio (HR) and tapped density, together with the overall assessment of the cleaning process of seed flowability, are listed in Table 3.

The Hausner ratio (HR) ranged from 1.0 to 1.11 for all the tested samples (before and after cleaning), except for the KWS Irina1 sample, which changed from the character of excellent to adequate after cleaning. Increased values of tapped density were observed in almost all studied varieties—after the cleaning process. With the constant weight, the particle density decreases—which is logical (debris fall of, abrasion).

As it is obvious from Table 3, that flowability remains *“excellent”* for all the samples after the cleaning process, except the variety KWS Irina1. For the variety KWS Irina1, the cleaning process led to worsening flowability according to both parameters—HR and CI, and contrarily, the values of AOR shows a decrease of about 32%—thus, flowability improved to “excellent.” These results imply nonuniformity of methods used—the least sensitive is assessed according to AOR (no flowability difference is shown if either value decreases by about 35% between the cleaned and uncleaned samples). In order to show the differences between the varieties, the solution could be to split up the area “excellent” into three further subareas, for example, values in the 20–24 range as *super excellent* flowability character, the 15–19 range as *ultra excellent,* and values below 15 as *ultra exemplary* flow. This would increase the method's sensitivity.

### 3.3 Effect of the cleaning process on barley seed friction parameters

**Figure 6:**
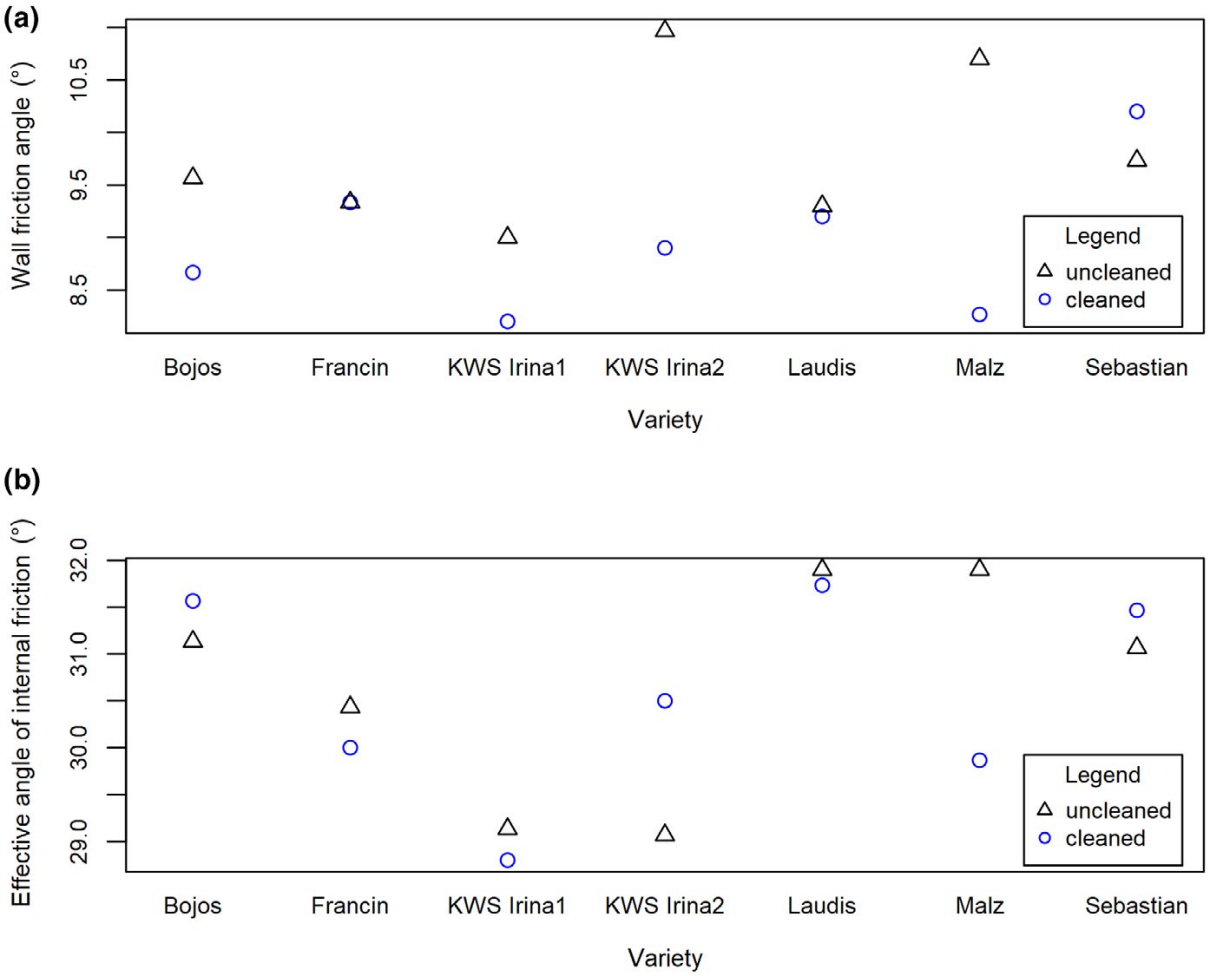
Mean values of (a) WFA and (b) AIF[E] depending on the barley variety and cleaning

Figure 6 shows a difference in the mean of the angle of the wall friction (Figure 6a) and the angle of the internal friction (Figure 6b) between the cleaned and uncleaned samples of the particular barley varieties.

Figure 6a clearly shows that the change of WFA differs among the cleaned and uncleaned samples more than among the particular barley varieties; the samples had distinctly lower WFA after the cleaning process, except for the Francin and Laudis varieties—where no significant difference was observed for cleaned and uncleaned samples. In the case of the Sebastian variety, the cleaned samples had an even higher WFA. KWS Irina2 and Malz—both uncleaned—had the highest WFA values, while the lowest values were measured for the samples of KWS Irina1 and Malz—both cleaned. Figure 6b shows that AIF[E] is not affected by the cleaning process so clearly—some varieties (Bojos, KWS Irina2, Sebastian) had higher AIF[E] after the cleaning process, while the other varieties had lower AIF[E] when uncleaned. Moreover, the values of AIF[E] differ mainly among the varieties. Laudis and Maltz—both uncleaned—had the highest AIF[E]; by contrast, KWS Irina1 cleaned and KWS Irina2 uncleaned had the lowest values.

Lower values of the internal friction angle for uncleaned samples of some varieties could imply that the uncleaned material contains fine dust or other fine impurities that contribute to reducing friction.

The wall friction angle is greater for samples before the cleaning process. From the point of view of flow, the same conclusion was reached as measured by densities, that is, the degree of purity of the barley received in the tested cases does not significantly affect their flow. In this sense, the same conclusion was reached as in the HR and CI experiments.

The results of two‐way ANOVA are displayed in Table 4. The results imply that the barley variety had the main effect on the AIF[E], while the cleaning had no significant effect. This is in line with the hypothesis presented above. A significant effect of the interaction of the variety and cleaning on the AIF[E] was also found. This shows that the effect of specific varieties on the AIF[E] differs depending on the cleaning.

**Table 4:** The results of two‐way ANOVA

		*df*	Sum Sq	Mean Sq	*F* value	Pr(>F)	Significance
AIF[E]	variety	6	36.29	6.048	10.926	2.89 × 10^−6^	**
	cleaning	1	0.10	0.105	0.190	0.6665	
	variety:cleaning	6	10.19	1.698	3.068	0.0195	*
	Residuals	28	15.50	0.554			
WFA	variety	6	8.147	1.358	2.643	0.037004	*
	cleaning	1	7.292	7.292	14.191	0.000782	**
	variety:cleaning	6	10.513	1.752	3.410	0.011841	*
	Residuals	28	14.387	0.514			

* *p* < .05

** *p* < .001.

In the case of WFA, cleaning was found to have the main effect, although the effect of the barley variety was significant as well. Moreover, a significant effect of the interaction of the variety and cleaning was observed.

### 3.4 Correlation analysis of physical parameters

**Figure 7:**
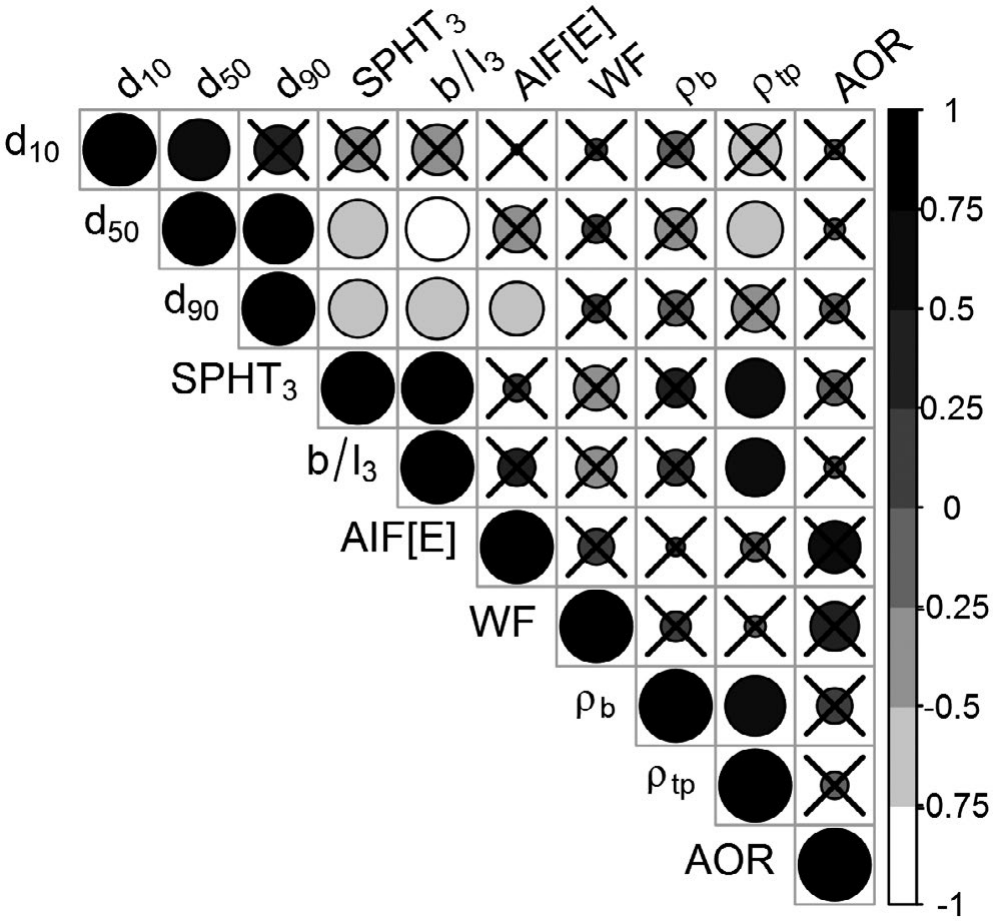
Correlation plot. Shades of gray indicate positive, negative, or zero correlation, while circle size signifies the strength of the correlation (Pearson's correlation coefficient). Crossed circles imply no significant correlation. d_10_, d_50_, d_90_–_10_,d_50_ resp. 90% particle representation for the detected size, SPHT_3_—sphericity, b/l_3_—ratio of width/length, AIF[E]—effective angle of internal friction, WFA—wall friction angle, ρ_b_—bulk density, ρ_tp_—tapped density, and AOR—angle of repose

The data of all the physical parameters were subjected to correlation analysis. The results—in the form of a correlation matrix—were then plotted (Figure 7).

As expected, a strong positive correlation was observed between d_50_ and d_10_, d_50_ and d_90_, SPHT_3_ and b/l_3_, SPHT_3_ and ρtp, b/l_3_ and ρ_tp_, and ρ_b_ and ρ_tp_. A strong negative correlation was observed between d50 and SPHT_3_, d_50_ and b/l_3_, d_50_ and ρ_tp_, d_90_ and SPHT_3_, d_90_ and b/l_3_, and d_90_ and AIF[E]. The negative correlation between the particle size d_90_ and the value of the effective angle of internal friction is interesting, for example, KWS Irina1—cleaned achieving values of d_90_ = 4.2 mm and AIF[E] =28.9°. From the positive correlation b/l_3_ and ρ_tp_, it is clear that particles with a higher proportion of b/l_3_, that is, with a more spherical particle shape, show a lower volume at vibrations arising, for example, during transport of the sample along the production line. One example may be the sample of Francin—cleaned b/l_3_ = 0.533 and ρ_tp_ = 778 kg/m^3^.

### 3.5 Linear regression of physical parameters

A linear regression analysis was performed for the significantly correlated pairs, with the outcomes presented in Table 5. A significant linear relationship was observed for all the tested pairs; nevertheless, the coefficient of determination was not strong.

**Table 5:** Results of linear regression analysis

Factors	*p*	*R* ^2^	Regression equations
AIF[E]–d_90_	.004	0.251	*y* = −7.314*x* + 60.847
b/l_3_–d_90_	.003	0.485	*y* = −0.098*x* + 0.925
SPHT_3_–d_90_	.012	0.371	*y* = −0.106*x* + 1.156
b/ l_3_–d_50_	.001	0.550	*y* = −0.139*x* + 1.020
SPHT_3_–d_50_	.010	0.387	*y* = −0.144*x* + 1.240
ρ_tp_–d_50_	.027	0.293	*y* = −199.77*x* + 1,448.57
ρ_tp_–SPHT_3_	.011	0.378	*y* = 1,011.41*x* ‐ 1.48
ρ_tp_–b/ l_3_	.012	0.373	*y* = 1,209.94*x* + 99.07

## 4 CONCLUSION

In the present study, the influence of the barley cleaning process in relation to physical properties was investigated for seven barley varieties.

Results showed that the cleaning process slightly increased barley grain sphericity as well as width‐to‐length ratio. Thus, higher grain homogeneity was achieved. Aforementioned parameters could be used for the relatively easy and rapid characterization of barley grains in practice.

From a flowability point of view, the results implied that the degree of cleaning (purity) does not have a significant influence on the flow properties of barley grains.

A significant influence was observed arising from mutual interactions of the barley variety and AIF[E], where the values of AIF[E] varied depending on the barley variety, while the cleaning process did not significantly affect the values of AIF[E]. On the other hand, in the case of wall friction angle (WFA), the main influence was in the cleaning process rather than the variety. Nevertheless, the effect of variety on the values of WFA was also significant.

The values of WFA were greater for precleaning samples than for postcleaning samples. The lower wall friction angle value after mechanical cleaning facilitates the movement (flow) of the cleaned grains over the contact material within the processing line. This is also confirmed by the lower energy loss during material transport after the cleaning process within the production line. It is also desirable to provide a suitable structural contact material with a varying wall friction angle of the particles.

## CONFLICTS OF INTEREST

The authors declare no conflict of interest.

## ETHICAL STATEMENT

This study does not involve any human or animal testing.
